# The antihyperglycemic effect of pulsed electric field-extracted polysaccharide of *Kaempferia elegans* officinale on streptozotocin induced diabetic mice

**DOI:** 10.3389/fnut.2022.1053811

**Published:** 2022-12-07

**Authors:** Huan-Qing Lei, Dong-Mei Li, Meng-Wai Woo, Xin-An Zeng, Zhong Han, Ruo-Yong Wang

**Affiliations:** ^1^School of Food Science and Engineering, South China University of Technology, Guangzhou, China; ^2^Guangdong Key Laboratory of Ornamental Plant Germplasm Innovation and Utilization, Environmental Horticulture Research Institute, Guangdong Academy of Agricultural Sciences, Guangzhou, China; ^3^Department of Chemical and Materials Engineering, University of Auckland, Auckland, New Zealand; ^4^Department of Food Science, Foshan University, Foshan, Guangdong, China; ^5^Preparatory Office of Yangjiang Applied Undergraduate College, Yangjiang, China; ^6^Overseas Expertise Introduction Center for Discipline Innovation of Food Nutrition and Human Health (111 Center), Guangzhou, China; ^7^Guangdong Provincial Key Laboratory of Intelligent Food Manufacturing, Foshan University, Foshan, China; ^8^Air Force Medical Center People’s Liberation Army, Beijing, China

**Keywords:** *Kaempferia elegans* polysaccharide, streptozotocin induced diabetic mice, gluconeogenesis, fasting blood glucose, enzyme activity

## Abstract

*Kaempferia elegans* polysaccharide (KEP) was extracted using a high-voltage pulsed electric field-assisted hot water method. Its physicochemical properties, *in vitro* activity and hypoglycemic effect was investigated. Experiments were undertaken with diabetic mice models and the potential mechanism of KEP to improve blood glucose levels was unveiled through measurements of relevant indicators in the serum and liver of the mice. Results showed that KEP is mainly composed of glucose, rhamnose, arabinose, and galactose. It has certain DPPH and ABTS free radical scavenging ability and good α-glucosidase inhibitory ability, indicating that KEP has the potential to improve blood glucose levels in diabetes patients. The experimental results of KEP treatment on mice showed that KEP could control the continuous increase of fasting blood glucose levels. The potential mechanisms behind this blood glucose level control composes of (1) increasing the glucokinase and C peptide levels and decreasing Glucose-6-phosphatase content for improving key enzyme activity in the glucose metabolism pathway. This promotes the consumption of blood glucose during glycolysis, thereby inhibiting the production of endogenous glucose in gluconeogenesis pathway; (2) reducing triglyceride, total cholesterol, low density lipoprotein cholesterol, and increasing high density lipoprotein cholesterol content, for regulating blood lipid indicators to normal levels; and (3) by improving the activities of catalase, glutathione peroxidase, and antioxidant enzymes superoxide dismutase for further improving the antioxidant defense system in the body to reduce blood glucose.

## Introduction

The irrational lifestyle and unbalanced diet structure of modern society, underpinned by high sugar and fat intake, have led to diabetes becoming one of the major chronic diseases. In 2019, the International Diabetes Federation (IDF) reported that approximately 463 million adults have diabetes mellitus with 4.2 million deaths (1). Expert are predicting that the number of people with diabetes mellitus will rise to 700 million by 2045 (2). Diabetes is clinically classified into type 1 diabetes mellitus, type 2 diabetes mellitus, and gestational diabetes mellitus (3). It is thought that half of the world’s population is undiagnosed because some of the symptoms of type 2 diabetes are not obvious and the patients have become younger. Diabetes has become a hidden killer in modern society, especially in the elderly, as it often leads to a range of complications, such as eye and heart disease and kidney failure. Currently, insulin and some various oral hypoglycemic agents such as glibenclamide, metformin, sulfonylureas, and α-glucosidase inhibitors have been the primary treatment for diabetes mellitus. These drugs are, however, expensive and some of them have serious side effects, including hypoglycemia, liver problems, drug resistance, and so on (4–6). Therefore, the development of plant-derived biologics to lower blood glucose is of great value and significance.

Polysaccharides are biological macromolecules that are composed of multiple monosaccharides as the primary building blocks, and some plant polysaccharides in particular have been shown to exhibit antioxidant (7), hypoglycemic (8–11), immune regulatory, and other physiological activities with potential applications in food and pharmaceutical industries. It has been shown that red bean polysaccharide, cowpea polysaccharide (12), lucidum polysaccharides (13, 14), pumpkin polysaccharide (15), bitter gourd polysaccharide (16), *Elaeagnus angustifolia* polysaccharide (17) all have hypoglycemic effects. Studies reported that the main mechanisms of polysaccharide hypoglycemia include promoting insulin secretion; repairing damaged islet tissues (e.g., islet β cells); regulating glucose metabolism pathways such as gluconeogenesis, glycolysis, and pentose phosphate pathway key enzyme activity; improving antioxidant capacity of the body, inhibiting inflammation, enhancing cell glucose uptake capacity; and relieving lipid metabolism disorder and symptoms caused by hyperglycemia (18). Thus, some plant polysaccharides can be explored as a novel agent for the treatment of diabetes mellitus.

*Kaempferia galanga* (*K. galanga*) is an aromatic herb grown mainly in tropical and subtropical Asia (19) and is used in the treatment of cold, dry cough, toothaches, rheumatism, hypertension, and so on (20). Modern pharmacological investigations have revealed that the extracts and natural products identified from *K. galanga* exhibited comprehensive bioactivities, including antitumor, antioxidant, anti-inflammatory, and other health effects (21, 22). Studies on water-soluble polysaccharides extracted from *K. galanga* (KGPs) showed that fucose, arabinose, xylose, rhamnose, mannose, galactose, glucose, glucuronic acid, and galacturonic acid were the main components of KGPs (23). *In vivo* antitumor test showed that KGPs effectively protected the thymus and spleen of tumor-bearing mice from attack and enhanced cellular immunomodulation, finally leading to tumor suppression (24). In addition, it has been widely used as a spice since its highly aromas. Among the *Kaempferia* species, the one with lavender flowers is *Kaempferia elegans (Wall.) Bak* (*K. elegans*) (25). It is also a perennial herb with aromatic rhizomes and a pungent flavor. It is extremely valuable for exploitation because it is easy and fast to cultivate. Although extensive pharmacological studies have been conducted on *K. galanga*, little work has been done on *K. elegans*. Particularly, there are almost no studies on *K. elegans* polysaccharides.

Pulsed electric field is a non-thermal processing technology (26). The effect of electric field can induce plant cells electroporation and increase their permeability, which accelerates the diffusion of extracts of the cells leading to enhanced extraction efficiency (27). Li et al. (28) used pulsed electric field assisted hot water to extract *Morinda citrifolia L.* (Noni) polysaccharide. Under suitable PEF conditions, the polysaccharide extraction yield reached 11.13%, which was significantly higher than that of the hot water extraction method. The PEF-assisted extracted polysaccharides also exhibited better antioxidant activity and anti-proliferative capacity than those under hot water extraction conditions. Liu et al. (29) studied the chemical structure characterization, antiproliferative, and antitumor activities of a *Morchella esculenta* polysaccharide (MEP) extracted by pulsed electric field. The results showed a polysaccharide extraction rate of 56.03 μg⋅mL^–1^ and a preliminary effect of the major polysaccharide (M2) in MEP on HT-29 cells *in vitro*, with potential therapeutic use in chemotherapy.

In the present study, we extracted *K. elegans* crude polysaccharide (KEP) using a pulsed electric field-assisted hot water method, studied the physicochemical properties, *in vitro* activity and hypoglycemic effect of KEP. We investigated the hypoglycemic effect of KEP on streptozotocin-induced type 2 diabetic mice by measuring body weight, fasting glucose, glucose metabolism, lipid levels, and oxidative stress. As far as we know, the present study is the unprecedented report on *K. elegans* crude polysaccharide and provides a pathway for further research on the use of *K. elegans* polysaccharide for effective treatment of diabetes mellitus.

## Materials and methods

### Materials

*Kaempferia elegans* rhizomes were collected from the garden of the environmental horticulture research institute (23°23′N, 113°26′E) at the Guangdong Academy of Agricultural Sciences, Guangzhou, China. Male Kunming mice (5-week old, body weight 16–20 g) were purchased from the Hunan Slake Jingda Co., Ltd. (Changsha, China). Phenol, trifluoroacetic acid, trichloroacetic acid, potassium sulfate, and gelatin were purchased from Shanghai Aladdin Biochemical Technology Co., Ltd. (Shanghai, China). Ethanol, chloroform, nbutanol, sulfuric acid, and hydrochloric acid were purchased from Guangzhou Chemical Preparation Plant. Streptozotocin (STZ), metformin hydrochloride, glucose, and sodium citrate were purchased from Shanghai Aladdin Biochemical Technology Co., Ltd. (Shanghai, China). Reagent kits for the determination of the glucokinase (GCK), Glucose-6-phosphatase (G6-P), C peptide (C-P), triglyceride (TG), total cholesterol (TC), high density lipoprotein cholesterol (HDL), low density lipoprotein cholesterol (LDL), superoxide dismutase (T-SOD), catalase (CAT), glutathione peroxidase (GSH-Px) were from Jiancheng Bioengineering Institute (Nanjing, China).

### Extraction of *Kaempferia elegans* polysaccharide

The rhizomes of *K. elegans* were washed, dried by hot air at 45°C, crushed with a universal high-speed crusher and then sieved to obtain the dried powdered form of the rhizomes. The powder was mixed with deionized water at a solid-liquid ratio of 1:18 and subjected to pulsed electric field treatment (electric field intensity of 31 kV/cm and number of pulses of 64). The model number of the pulsed electric field equipment is PEF-SY-500 (Guangzhou Paihu Technology Co., Ltd, Guangzhou, China). Thereafter, after hot water extraction at 98°C for 2.5 h, the supernatant was collected by centrifugation at 3000 rpm for 15 min, concentrated by rotary evaporator, and precipitated with 95% ethanol at 4°C (28). The precipitate was collected by centrifugation at 5000 rpm for 20 min, dried and redissolved with appropriate amount of deionized water, and the extract was frozen to remove the proteins by Sevage method to obtain the *K. elegans* crude polysaccharide (KEP).

### Analysis of *Kaempferia elegans* polysaccharide

The polysaccharide content of KEP was examined using phenol-sulfuric acid method (30), protein content was analyzed with the Coomassie brilliant blue kit. The total phenol content was determined using the Folin ciocalteu assay with gallic acid as the standard. The sulfate content in the polysaccharide was determined with the barium sulfate turbidimetry method (31). The uronic acid content was determined from the sulfuric acid-carbazole colorimetry (32) and the molecular weight of KEP was evaluated by gelpermeation chromatography (33), while the monosaccharide composition was analyzed by high performance anion exchange chromatography (ICS-5000, Dionex Inc., USA) (34). The KEP sample solution was scanned with a UV spectrophotometer in the range of 190–400 nm with a scan interval of 0.5 nm. A certain amount of KEP sample was weighed and scanned in the wave number range of 400–4000 cm^–1^ using the KBr compression method and the infrared spectra were obtained using a Fourier infrared spectrometer. A scanning electron microscope (SEM) was used to observe the surface microstructure of the freeze-dried sample KEP (35). The polysaccharide samples were sprayed with gold under vacuum and the morphological images were observed using scanning electron microscopy.

### Exploration of the *in vitro* activity of *Kaempferia elegans* polysaccharide

To evaluate the *in vitro* antioxidant activity of KEP, its DPPH, ABTS radical scavenging ability was assayed. The scavenging ability of DPPH and ABTS radicals was assessed spectrophotometrically. A certain amount of polysaccharide solution was mixed with DPPH solution or ABTS working solution, and the absorbance values of the solutions were measured at 517 and 732 nm, respectively, after the reaction for 30 min at room temperature and protected from light (36, 37).

The hypoglycemic activity of KEP *in vitro* was measured by assessing the α-glucosidase inhibition capacity. A certain amount of KEP solution with different concentrations was mixed with α-glucosidase and activated in a 37°C water bath for 10 min, then p-nitrophenyl-α-D-glucopyranoside (pNPG) substrate was added and mixed in a 37°C water bath for 20 min, and finally the reaction was terminated by the addition of sodium carbonate solution. The absorbance of the solution at 405 nm was measured after mixing (38, 39).

### Establishment of diabetic mice model

In all the experiments, the mice were housed in a negative pressure laboratory at 20–24°C (relative moisture: 50–70%) with a light-dark cycle (12/12 h). Firstly, the mice were required to undergo 7 days of acclimatization and a 12-h starvation period before the experiment, allowing free access to water. Experimental approval for the care and use of experimental animals was obtained from the animal ethics committee of South China University of Technology, animal ethics number 202508. Forty mice were randomly selected and injected intraperitoneally with 130 mg/kg ⋅ bw (body weight) of streptozotocin (STZ) solution to induce type 1 diabetes mellitus, while the same amount of normal saline was administered intraperitoneally to 10 mice. After 5 days, the mice were fasted for 12 h before measuring blood glucose level, and blood samples were collected from the tail on the sixth day to assess fasting blood glucose values and to observe the model.

### Experiment design and procedure

**FIGURE 1:**
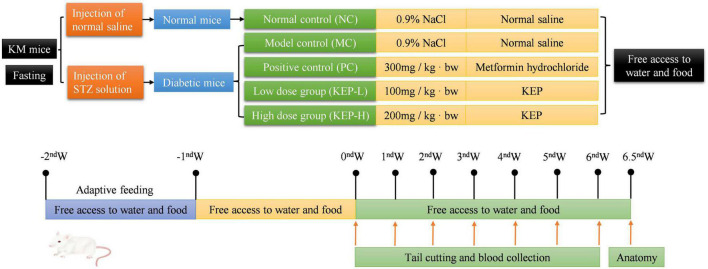
Animal experimental protocol and design.

The animal modeling, dosing and grouping protocols are shown in Figure 1. Fasting blood glucose values higher than 11.1 mmol/L were used as an indicator of successful induction in diabetic mice. The successful model mice were randomly divided into four groups of nine mice each, and nine normal mice were randomly selected as the normal group control. The polysaccharide fraction was KEP solution for the low-dose group (KEP-L: 100 mg/kg ⋅ bw) and the high-dose group (KEP-H: 200 mg/kg ⋅ bw); the positive control group (PC) was gavaged with a dose of 300 mg/kg ⋅ bw metformin hydrochloride; the normal group (NC, non-diabetic mice) and the model group (MC, diabetic mice) served as blank controls and were gavaged with equal amounts of saline.

All the experimental groups were intragastrically administered at the same time every day and the basic situation of mice in each group was observed daily from three aspects: mental state and activity, fur color and gloss, and cage cleanliness. The fasting blood glucose of mice in each group was measured by blood glucose meter at the beginning of the experiment and at the same time every week (tail cutting blood was collected at day 7, 14, 21, 28, 35, and 42), while the body weight was recorded every 48 h.

At the end of the sixth week, the mice were sacrificed by cervical dislocation and the orbital blood was collected in capillary tubes. The light yellow supernatant from the orbital blood was collected after centrifugation at 2500 rpm for 20 min. The pancreas, liver, kidney, and spleen were also excised quickly, blotted dry, weighed, and the organ index was calculated according to the following equation:


(1) Organ  index=organ  weightbody  weight

The liver and kidney tissues were homogenized with a biospecimen homogenizer under ice-cold saline solution, and the supernatant was collected by centrifugation at 3000 rpm for 20 min and used for subsequent analysis.

### Effects of *Kaempferia elegans* polysaccharide on glucose metabolism, lipid levels, and oxidative stress-related enzymes in diabetic mice

#### Determination of glucose metabolism related enzymes

The contents of glucokinase (GCK) and glucose-6-phosphate (G6Pase) content in the liver and the C-peptide content in the serum were determined by enzyme-linked immunosorbent assay kit.

#### Determination of blood lipid profile

The content of triglyceride (TG) and total cholesterol content (TC) (16) in mice serum were determined with the GPO-PAP method within the range of 0–9.04 mmol/L, and with the COD-PAP method within the range of 0–10.34 mmol/L, respectively. The amount of high density lipoprotein-cholesterol (HDL-c) and low density lipoprotein-cholesterol (LDL-c) were determined by homogeneous direct assay kits.

#### Determination of oxidative stress related enzyme activity

The activity of superoxide dismutase (T-SOD) and catalase activity (CAT) in mouse liver were determined by xanthine oxidase and ammonium molybdate methods, respectively, while the activity of glutathione catalase was determined by the assay kit according to the manufacturers specification (40).

### Statistical analysis

All the analyses were conducted in triplicates and the results were expressed as mean ± standard deviation (SD). Microsoft Excel 2019, ELISA calc, SPSS version 25 and Origin Pro version 9.1 were used for experimental data processing. Analysis of variance (ANOVA) was employed separating means at *p* < 0.05 using the Duncan multiple test.

## Results and discussion

### Composition of *Kaempferia elegans* polysaccharide

**FIGURE 2:**
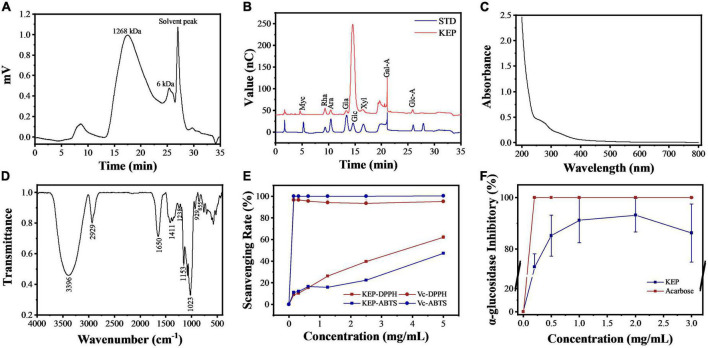
**(A)** Molecular weight of KEP. **(B)** Ion chromatogram spectra of crude polysaccharide (KEP) and monosaccharide mixture (STD). **(C)** UV spectrum analysis of KEP. **(D)** IR spectrum analysis of KEP. **(E)** Antioxidant activity of KEP. **(F)** Inhibition of α-glucosidase by KEP. Each value was represented as mean SD of three independent experiments.

The extraction rate was 405.04 mg/g under the pulsed electric field intensity of 31 kV/cm, pulse number of 64, extraction temperature of 98°C, extraction time of 2.5 h, and solid-liquid ratio of 1:18. The extraction rate of polysaccharides by pulsed electric field assisted extraction was 53% higher than that by hot water extraction. The GPC profile of KEP (Figure 2A) showed that the molecular weight of one of the KEP subgroups was around 6 kDa, while the main fraction was concentrated at 1268 kDa.

As shown in Table 1, the total carbohydrate content of the crude polysaccharide was 88.8 ± 0.9%, which was similar to a previous report by Dwl et al. (41) with 0.5% protein, as it was only preliminarily purified. Derivatives with hydroxyl groups replaced by sulfuric acid groups in polysaccharides have been reported to exhibit prominent antiviral activity (34), thus, the sulfate content in KEP was determined as 27.86 ± 0.91 mg/g, with total uronic acid content of 75.54 ± 11.08 mg/g. Furthermore, the ion chromatograms of the crude polysaccharide and standard compound (Figure 2B) indicated that KEP is composed of six monosaccharides with glucose accounting for 84.44%, followed by rhamnose, arabinose, galactose, xylose, and fucose at 6.52, 3.38, 2.26, 1.97, and 1.41%, respectively, suggesting that KEP was a glucose-based polysaccharide, consistent with the main monosaccharide composition of polysaccharide report (42).

**TABLE 1:** Chemical composition and monosaccharide composition of KEP.

Composition	Content (mg/g Crude polysaccharide)	Monosaccharide composition (g)	Mole percentage (%)
Total sugar	887.73 ± 8.73	Fucose	1.41
Protein	4.95 ± 1.10	Rhamnose	6.52
Total polyphenol	3.71 ± 1.20	Arabinose	3.38
Sulfate radical	27.86 ± 0.91	Galactose	2.27
Uronic acid	75.54 ± 11.08	Glucose	84.44
		Xylose	1.97

A full-band scan of the KEP solution using a UV spectrophotometer showed a small absorption peak at 280 nm as shown in Figure 2C, indicating that the KEP contained a small amount of protein, which is consistent with the protein assay results; the deproteinization step has removed most of the protein. Figure 2D shows the infrared spectrum of KEP with a full band scan at 400–4000 cm^–1^. The two large peaks at 3396 cm^–1^ and 2929 cm^–1^ in the figure are the stretching absorption peak of hydroxyl group and the absorption peak of alkyl carbon-hydrogen bond (35), both characteristic of sugars. 1650 cm^–1^ represents the stretching vibration of carbon-oxygen double bond and 1411 cm^–1^ is the stretching vibration of carbon-carbon double bond, indicating the presence of glyoxalate in the polysaccharide, which is consistent with the previous results of glyoxalate detection. The absorption peak at 1238 cm^–1^ may be the stretching vibration of the oxygen-sulfur bond, indicating the presence of sulfate group in the polysaccharide, but the absorption peaks are both relatively small, indicating a low content, which is consistent with the previous results of sulfate group assay; in addition, the peaks at 855 cm^–1^ and 929 cm^–1^ indicate that the polysaccharide has α-glycosidic bond and the presence of α-glycoside (43).

The SEM images of the KEP are shown in Supplementary Figures 1a,b. The polysaccharide is mainly composed of spherical particles accumulated in flakes with the surface displaying obvious honeycomb-like large holes. Supplementary Figure 1c shows that the polysaccharide structure is loose, and a large number of spherical particles are connected by filamentous polysaccharides to form a loose and porous structure.

### Exploration of the *in vitro* activity of *Kaempferia elegans* polysaccharide

The DPPH and ABTS radical scavenging ability of KEP is shown in Figure 2E. The DPPH radical scavenging capacity increased proportionally with increasing KEP concentration, indicating that the DPPH radical scavenging capacity of KEP has a dose effect, reaching 62% at 5 mg/ml with an IC50 value of 3.71 mg/ml. This is similar to the DPPH radical scavenging capacity of polysaccharides from ginger leaves and stems extracted by Chan et al. (44). The scavenging rate of ABTS radicals increased slowly with the increase of KEP concentration in the concentration range of 0.1–1.25 mg/ml, while the scavenging rate started to increase in the range of 1.25–5 mg/ml. This indicates that the polysaccharide has limited ability to capture ABTS free radicals and has obvious antioxidant ability only at a certain concentration.

By inhibiting α-glucosidase activity, glucose production can be delayed and postprandial blood glucose values can be lowered or fasting blood glucose values can be regulated, thus controlling the diabetic condition. The *in vitro* hypoglycemic effects of the KEP were investigated *via*α*-*glucosidase inhibitory activity assays using acarbose as a positive reference. As shown in the Figure 2F, the inhibition of α-glucosidase by KEP at 2 mg/ml reached 91.47% with increasing concentration, indicating that the inhibition of KEP was effective and highly dose-dependent. *In vitro* antioxidant assay of KEP shows its potential to improve blood glucose in diabetic patients.

### Effects of *Kaempferia elegans* polysaccharide on body weight and organ weight

**FIGURE 3:**
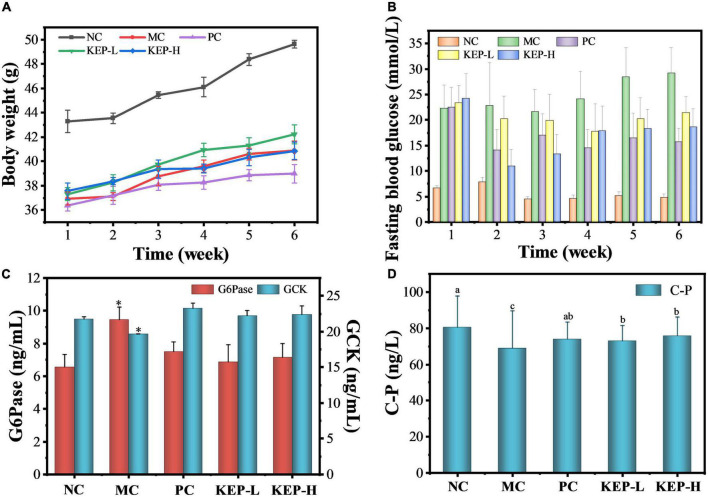
**(A)** Changes in the body weight of normal and diabetic mice models. **(B)** Changes in fasting blood glucose of normal and diabetic mice models. **(C)** Effects of different treatments on the GCK and G6Pase of normal diabetic mice models. **(D)** Effects of different treatments on urea nitrogen and C-peptide contents of normal diabetic mice models. ^a,b,c^Values with different letters are significantly different (*p* < 0.05). *Value is significantly different from those of other groups in the same column (*p* < 0.05). Each value was represented as mean ± SD of three independent experiments.

Injecting 130 mg/kg-bw of STZ to mice, the success rate of the model after 5 days was high, reaching more than 95%. The effects of KEP on the body weights of diabetic mice is presented in Figure 3A, and data shows that the body weight of all mice increased within 6 weeks of intervention, while the body weights of the diabetic mice were significantly lower than the body weights normal mice. The increase of body weight indicated that all the mice were in the growth period, and the reduced weights of the diabetic mice indicated that diabetes can significantly reduce the body weight, which is consistent with the reported symptoms of weight loss in diabetic mice (45). During the intervention, the body weight of normal mice increased steadily from 43.29 to 49.62 g and was significantly higher than other groups. The weight of the positive group became significantly lower than the other groups during the fourth week and reached 38.99 g in the sixth week, attributed to the adverse effects of metformin hydrochloride (46). Compared with the model control group (MC), the body weight of mice in the two groups treated with polysaccharide increased at different degrees, but there was no significant difference in weight between the two groups.

The data for the organ weight was presented in Table 2. The results showed that the organ index of liver and kidney in the model control group (MC) were significantly higher than the normal control group (NC), indicating that the liver and kidney in the model control group were swollen and seriously damaged. Moreover, by comparison with the liver and kidney organ indexes of the normal control group (NC), the liver and kidney organ indexes of the other three groups, including the positive control group (PC), the low-dose group (KEP-L), and the high-dose group (KEP-H), were also significantly increased. However, the liver and kidney organ indexes in these three groups were significantly lower than the indexes in the model control group.

**TABLE 2:** Changes in the organ indexes of normal and diabetic mice models.

Group	Organ index (%)			
	
	Liver	Kidney	Spleen	Pancreas
NC	3.98 ± 0.29^a^	1.31 ± 0.12^a^	0.22 ± 0.02^a^	1.78 ± 0.14^a^
MC	5.51 ± 0.31^c^	1.83 ± 0.11^c^	0.26 ± 0.08^a^	1.11 ± 0.04^b^
PC	4.91 ± 0.03^b^	1.64 ± 0.01^b^	0.24 ± 0.05^a^	1.30 ± 0.05^b^
KEP-L	4.63 ± 0.48^b^	1.59 ± 0.10^b^	0.20 ± 0.06^a^	1.27 ± 0.31^ab^
KEP-H	4.50 ± 0.17^b^	1.56 ± 0.18^b^	0.18 ± 0.02^a^	1.23 ± 0.14^b^

^a,b,c^Values with different letters are significantly different (*p* < 0.05).

The normal functioning of the liver and kidneys supports many important functions of life activity. The results indicated that both positive drugs and polysaccharides played a role in repairing organs during the intervention. There were also no significant differences in spleen organ index among different groups. For the pancreatic organ index, it was significantly decreased by 1.11% in the model control group when compared to the positive normal group. The higher organ indexes of the positive control group and the polysaccharide group, when compared to the indexes of the model control group, indicated the promising mitigating effects of KEP on pancreatic injury.

### Effects of *Kaempferia elegans* polysaccharide on fasting blood glucose levels

Fasting blood glucose measurement is considered the most direct indicator for monitoring diabetes (45) and often represents the basic insulin secretion function and role. Human normal fasting blood glucose ranges between 3.9 and 6.1 mmol/L, and levels higher than 11.1 mmol/L can be diagnosed as diabetic. Mice fasting blood glucose concentrations above 11 mmol/L were also considered to be diabetic. Figure 3B shows a fluctuating trend in the fasting blood glucose values of mice in each group with large fluctuations observed in the group of mice with diabetic symptoms, compared with the smaller range of fluctuation in the group of normal mice. The fasting blood glucose of the normal mice were maintained at a low level (4.60–7.86 mmol/L), while the fasting blood glucose of the mice in the model group continued to increase, indicating aggravation of the disease. Significant differences were observed between the model control group and the positive control group from the fourth week, and the positive drugs played a significant role in reducing blood glucose, while the levels in the model control group reached the maximum value of 29.19 mmol/L at the sixth week. In addition, the fasting blood glucose values of the polysaccharide group and the positive control group decreased after the second week of gavage, indicating that KEP and metformin hydrochloride showed obvious hypoglycemic effects. However, there was a rebound in the fasting blood glucose value after the third week to fluctuate in the range of 15–22 mmol/L, with no significant differences between the groups. Specifically, metformin hydrochloride acted rapidly but mainly stabilized blood glucose in later period, while the low dose of crude polysaccharide mainly stabilized blood glucose at a slower rate. On the other hand, the high doses of crude polysaccharides acted rapidly and performed even better when compared with the positive control. At the latter stages, the rate remained stable, suggesting that KEP exhibited similar effects as metformin hydrochloride in controlling and stabilizing blood glucose.

### Effects of *Kaempferia elegans* polysaccharide on glucose metabolism related enzymes

Glycolysis pathway is an important component of the overall glucose metabolism, converting glucose into energy. Glucokinase (GCK) is a hexokinase isoenzyme that catalyzes glucose phosphorylation in the liver of mammals and plays the role of “glucose sensor” in the regulation of blood glucose, because its synthesis is mainly induced by insulin which can provide timely feedback for regulating the synthesis and secretion of insulin. When the glucose concentration in the body increases, GCK can also control the reaction rate of glucose oxidative decomposition for promoting the generation of liver glycogen and maintaining the dynamic balance of blood glucose (47, 48). Figure 3C shows that the GCK content of the model control group (19.67 ng/mL) was significantly reduced when compared with the normal control group, indicating reduced utilization of blood glucose. At the same time, the GCK content after the intervention of the KEP (22.24 ng/mL) was higher when compared with the normal control group (21.78 ng/mL), indicating that KEP can promote blood glucose consumption and reduce blood glucose level by increasing GCK content. The GCK content in the positive control group reached 23.25 ng/mL, but there was no significant difference among the normal control group, positive control group, low-dose group and the high-dose group. Thus, the GCK regulating effect of KEP was not superior to that of the positive drug.

The effect of KEP on the content of G6Pase is also presented in Figure 3C. It can be seen that the activity of Glucose-6-phosphatase (G6Pase) in the normal control group was 6.55 ng/mL, while that of the model control group significantly increased to 9.45 ng/mL. The activity of G6Pase were 6.87 and 7.14 ng/mL after the intervention of KEP, and with the exception of the model group, the activity of G6Pase in the other groups were in the normal state. This indicated the potential of the KEP for reducing the activity of G6Pase and the production of endogenous glucose which could also regulate the blood glucose level. The gluconeogenesis pathway is the only way for the body to synthesize monosaccharides during long-term starvation and G6Pase is one of the key enzymes in gluconeogenesis that can hydrolyze glucose-6-phosphate to produce glucose. It exists mainly in the liver, so its content directly affects the glucose content in the liver, and studies have shown that the expression levels of catalytic subunit and mRNA corresponding to G6Pase in the liver under diabetes typically increases to varying degrees. Therefore, the improved activity of G6Pase after the intervention of KEP may reveal the potential hypoglycemic mechanism of KEP (49).

Furthermore, C-peptide (C-P) is a connecting peptide secreted by pancreatic islet β cells that is mainly derived from its precursor proinsulin. Under the catalysis of carboxypeptidase, proinsulin can decompose into insulin and C-peptide with the same molecular weight, but C-peptide is not decomposed by the liver and does not react with insulin antibody and other substances. C-peptide can accurately reflect the pancreatic islet beta cell function of the body in addition to indirectly reflecting the insulin and pancreatic damage level of the body (50). In this study, the content of C-peptide (C-P) was determined to explore the insulin level of mice in each group after 6 weeks of gavage. Figure 3D showed that the C-P concentration in the normal control group was 90.74 ± 9.87 ng/L, while the C-P concentration in the groups with mice pancreatic islet β cells injury induced by STZ were significantly reduced. Particularly, the values for the model control group, KEP-L group and KEP-H group were 62.07 ± 1.25, 76.82 ± 6.21, and 77.29 ± 2.15 ng/L, respectively. The concentration of C-P in the model control (MC) group accounted for 68.4% of the normal control (NC) group, which means that the insulin secretion is the least and the pancreas is seriously damaged. The concentration of C-P in the polysaccharide (KEP-H and KEP-L) group accounted for 84–85% of the normal control (NC) group, indicating that the crude polysaccharide intervention could improve the insulin level of the diabetic mice and repair the pancreas.

### Effects of *Kaempferia elegans* polysaccharide on blood lipid levels

**FIGURE 4:**
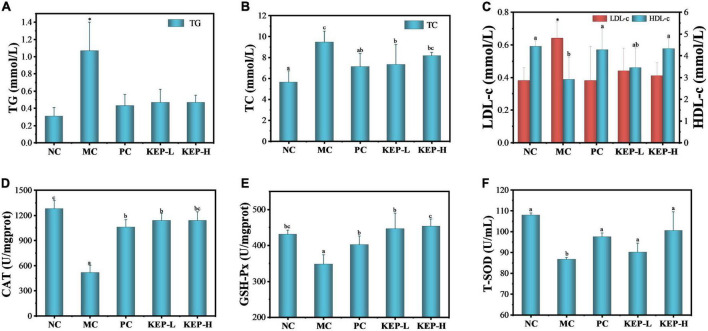
Effects of different treatments on lipid levels of normal and diabetic mice models: **(A)** TG, **(B)** TC, and **(C)** LDL-c and HDL-c. Effects of different treatments on the oxidative stress related enzymes of normal and diabetic mice models: **(D)** CAT, **(E)** GSH-Px, and **(F)** T-SOD. ^a,b,c^Values with different letters are significantly different (*p* < 0.05). *Value is significantly different from those of other groups in the same column (*p* < 0.05). Each value was represented as mean ± SD of three independent experiments.

Blood glucose and blood lipid are the two major metabolic indicators in the body. Abnormal blood lipid level in patients with diabetes which typically manifests in triglyceride (TG), cholesterol (TC), high-density lipoprotein cholesterol (HDL-c) and low-density lipoprotein cholesterol (LDL-c) (51), can lead to aggravation of the disease. Therefore, observing the changes of blood lipid levels in diabetic mice can aid in exploring the hypoglycemic mechanism of KEP. Figures 4A–C shows the effect of crude polysaccharide on TG, TC, LDL-c, and HDL-c. The TG and LDL-c values of the model control group were significantly higher than the normal control group, but the positive drugs metformin hydrochloride and KEP significantly reduced the TG and LDL-c levels in diabetic mice. Although the average values for the model control group was slightly higher, there was no significant difference between the model control group and the normal control group. The results suggested that KEP had significant reducing effects on TG and LDL-c, but had no considerable dose effects. With regards to the TC, the model control group presented significantly higher values than the normal control group, while the positive control group presented significantly lower values than the model control group which decreased by 24.8%. There was also no significant difference between the positive control group and the normal control group, while reductions of up to 22.55 and 13.80% were observed for KEP-L and KEP-H, respectively; there was no significant difference between the high-dose group and the model control group. Thus, in terms of reducing the TC value, the positive control group presented better results than the low dose group. For the HDL-c, the model control group presented significantly lower values than the normal control group, but the values for the other groups increased significantly than the normal control group. Particularly, the positive control group and the high dose group increased significantly by 47.2 and 49.0%, respectively, with only 19.0% increased observed for the low dose group. Overall, the TG, TC, LDL-c in the model control group increased significantly in terms of blood lipid levels when compared with normal control group, which was consistent with a previous report (51). The positive control group and the polysaccharide group presented different degrees of improvement, indicating that the blood lipid indexes in diabetic mice were indeed abnormal. However, the positive drugs metformin hydrochloride and KEP could improve and regulate blood lipid levels in diabetic mice after 6 weeks of continuous gavage.

### Effects of *Kaempferia elegans* polysaccharide on oxidative stress related enzyme activity

Hydrogen peroxide is considered a kind of metabolic waste in biological metabolic system since its oxidative ability and secondary product of hydroxyl radical can cause harm to the body (52, 53). Catalase (CAT) is a key enzyme in the biological defense system and can promote the decomposition of hydrogen peroxide in the body for removing excesses and protection from hydrogen peroxide toxicity. CAT exists in animal liver and its enzymatic activity strengthens the antioxidant system of the body, which is of great significance to metabolic activities (54). Glutathione peroxidase (GSH-Px) is one of the catalases that can specifically catalyze the reactions of GSH and hydrogen peroxide for effectively removing free radicals, and it is a common indicator for evaluating the antiperoxidation ability of the body. Superoxide dismutase (T-SOD) is also an important member of the biological antioxidant enzyme system and can catalyze the disproportionation of superoxide anion radicals to produce oxygen and hydrogen peroxide, while playing a vital role in the oxidation and antioxidant balance of the body (55). Figures 4D–F shows the effects of KEP on CAT, GSH-Px, and T-SOD in the oxidative stress system of diabetic mice. Compared with the normal control group, the activities of CAT, GSH-Px, and T-SOD in the model control group were significantly decreased, indicating that STZ-induced destruction of pancreatic islet β cells also damaged the antioxidant system of the mice. The low activity of the oxidative stress-related enzymes corresponded to serious oxidative damage in the body. The activities of CAT, GSH-Px, and T-SOD in the KEP groups increased with varying degrees, suggesting that KEP, like positive drugs, could enhance the antioxidant system enzyme activity. However, the polysaccharide group presented significantly lower CAT activity than the normal control group, and there was no significant difference with the positive control group, indicating that KEP can only partially promote CAT activity. The order of the activity of GSH-Px was polysaccharide group > normal control group > positive control group, indicating the potential of KEP for promoting the activity of GSH-Px, while the order of activity for T-SOD enzyme was normal control group > high dose group > positive control group > low dose group. However, there was no significant difference among the four groups in T-SOD enzyme activity, indicating that KEP can significantly improve T-SOD enzyme activity in normal mice. Generally, long-term hyperglycemia and hyperlipidemia environment in diabetics can lead to the generation of a large amount of reactive oxygen species that can keep the body in a state of oxidative stress. The oxidative stress state and the blood lipid state of hyperglycemia can affect each other and form a vicious circle (56). Therefore, KEP can be used as an effective antioxidant to intervene the oxidative stress caused by hyperglycemia, inhibit lipid peroxidation, scavenge free radicals and improve antioxidant capacity. This breaks the vicious cycle and improves blood glucose regulation.

## Conclusion

*Kaempferia elegans* polysaccharide is mainly composed of glucose, rhamnose, arabinose, and galactose. It has certain DPPH and ABTS free radical scavenging ability and good α-glucosidase inhibiting ability. The animal experiment results indicate that KEP was effective in improving the persistent increase in fasting glucose in diabetic mice, without side effects on the body weight. The organ indexes showed that the liver, kidney, and pancreas of STZ-induced diabetic mice were damaged to a certain extent, while KEP showed a potential repairing effect on the organs. The possible mechanisms of KEP for improving blood glucose in diabetic mice were observed from three aspects:

(1) Blood glucose regulation by increasing GCK and C-P content, reducing G6Pase, improving the content of key enzymes in glucose metabolism, promoting the consumption of blood glucose during glycolysis, inhibiting the production of endogenous glucose in gluconeogenesis and promoting the production of insulin.
(2) Preventing further deterioration of hyperlipidemia diabetes by reducing TG, TC, LDL-c content, increasing HDL-c content, and regulating blood lipid indicators to normal levels.
(3) Improving antioxidant defense system in the body for achieving hypoglycemic effect through good free radical scavenging ability and improving the activities of CAT, GAH-Px, and T-SOD.

Insights into the accurate mechanisms behind the glucose regulatory function of KEP will be required in the future, to illustrate the potential of KEP as a treatment agent for diabetes.

## Data availability statement

The original contributions presented in this study are included in the article/Supplementary material, further inquiries can be directed to the corresponding authors.

## Ethics statement

This animal study was reviewed and approved by the Animal Center of South China University of Technology.

## Author contributions

H-QL examined the antihyperglycemic effects of the *Kaempferia elegans* polysaccharides in mice. D-ML cultivated the *Kaempferia elegans*. H-QL and D-ML isolated and characterized polysaccharides from *Kaempferia elegans*. ZH and R-YW revised the manuscript. X-AZ and R-YW contributed to the design of the study and data interpretation. M-WW contributed to the writing of the article and the polishing of language. All authors contributed to the article and approved the submitted version.
