# Supplementary material for: The antihyperglycemic effect of pulsed electric field-extracted polysaccharide of Kaempferia elegans officinale on streptozotocin induced diabetic mice
Source: Front Nutr. 2022 Dec 7;9:1053811. doi: 10.3389/fnut.2022.1053811 (PMC9769402; doi:10.3389/fnut.2022.1053811)
Supplement: Supplementary file 1 [file Data_Sheet_1.docx]

Figure S1. (a)-(e) Morphology of KEP.
